# Hub genes associated with immune cell infiltration in breast cancer, identified through bioinformatic analyses of multiple datasets

**DOI:** 10.20892/j.issn.2095-3941.2021.0586

**Published:** 2022-07-13

**Authors:** Huanyu Zhao, Ruoyu Dang, Yipan Zhu, Baijian Qu, Yasra Sayyed, Ying Wen, Xicheng Liu, Jianping Lin, Luyuan Li

**Affiliations:** 1 State Key Laboratory of Medicinal Chemical Biology and College of Pharmacy, Tianjin Key Laboratory of Molecular Drug Research, Nankai University, Tianjin 300350, China; 2 Department of Physiology and Pathophysiology, School of Basic Medical Sciences, Capital Medical University, Beijing 100069, China

**Keywords:** Bioinformatics, breast cancer, multi-datasets analysis, immune cell infiltration, survival analysis

## Abstract

**Objective:**

The aim of this study was to identify hub genes associated with immune cell infiltration in breast cancer through bioinformatic analyses of multiple datasets.

**Methods:**

Nonparametric (NOISeq) and robust rank aggregation-ranked parametric (EdgeR) methods were used to assess robust differentially expressed genes across multiple datasets. Protein-protein interaction network, GO, KEGG enrichment, and sub-network analyses were performed to identify immune-associated hub genes in breast cancer. Immune cell infiltration was evaluated with the CIBERSORT, XCELL, and TIMER methods. The association between the hub gene-based risk signature and survival was determined through Kaplan–Meier survival analysis, multivariate Cox analysis, and a nomogram with external verification.

**Results:**

We identified 163 robust differentially expressed genes in breast cancer through applying both nonparametric and parametric methods to multiple GEO (*n* = 2,212) and TCGA (*n* = 1,045) datasets. Integrated bioinformatic analyses further identified 10 hub genes: CXCL10, CXCL9, CXCL11, SPP1, POSTN, MMP9, DPT, COL1A1, ADAMDEC1, and RGS1. The 10 hub-gene-based risk signature significantly correlated with the prognosis of patients with breast cancer. Moreover, these hub genes were strongly associated with the extent of infiltration of CD4+ T cells, CD8+ T cells, neutrophils, macrophages, and myeloid dendritic cells into breast tumors.

**Conclusions:**

Integrated analyses of multiple databases led to the discovery of 10 robust hub genes that together may serve as a risk factor characteristic of the immune microenvironment in breast cancer.

## Introduction

Breast cancer (BC) remains the most common female cancer in women and is associated with severely high mortality rates^1^. Together with traditional cancer risk factors, such as unregulated cell growth and apoptosis evasion^2,3^, immune-manipulating mechanisms are crucial characteristics of cancers^4^. Cancer cells can influence their immune microenvironment by exerting immunosuppressive signals, evading immune recognition, or fueling tumor-promoting inflammation, thereby driving cancer progression^5^. Comparison of the gene expression profiles of paired clinical samples of tumors and normal tissues is valuable for identifying differentially expressed genes (DEGs) that may have important roles in the modulation of the BC immune microenvironment.

The vast genomics databases and rapidly advancing bioinformatics tools provide opportunities to search for DEGs associated with the cancer immune microenvironment. A wide variety of RNA-Seq datasets for BC have been deposited in public databases, such as Gene Expression Omnibus (GEO) and The Cancer Genome Atlas (TCGA). These databases are valuable for the discovery of disease-associated genes. However, most gene expression profile databases are quite small^6,7^. For example, the GEO consists of a variety of datasets with 20–300 clinical samples each, which clearly cannot reflect the disease conditions at the population level^8^. Investigators have attempted to simultaneously use multiple datasets to identify hub genes in BC, with the caveat that reliance on limited sample sizes can lead to biased outcomes^9,10^. In the meantime, new data-mining methods have been developed to mine complicated clinical databases, and may have great potential if used in combination^11^. For instance, to take advantage of the small but diverse GEO datasets, the method of robust rank aggregation (RRA) has been proposed for integrated analyses of multiple datasets with decreased levels of noise, to overcome the heterogeneity inherent in each of the relatively small platforms^12^. Additionally, the nonparametric NOISeq R package has been shown to efficiently control false discovery in experiments with biological replicates^13^.

In this study, we demonstrated the utility of integrated analyses in multiple BC datasets. We assessed multiple GEO and TCGA databases for BC by using RRA-ranked parametric (EdgeR) and nonparametric (NOISeq) methods to discover robust DEGs. We examined the robust DEGs in the protein-protein interaction (PPI) network through GO and KEGG enrichment analysis and sub-network analysis. We identified 10 hub genes that together may represent a risk signature that not only correlates with BC prognosis but also may serve as a biomarker for the immune microenvironment in BC.

## Materials and methods

### Data collection and processing

The available public RNA-sequencing data for BC in TCGA cohort (http://xena.ucsc.edu/)^14^, comprising 1,045 patients (with overall survival > 30 days) with normalized gene expression data (FPKM) and clinical information, were included. In addition, the gene expression data in 10 microarray datasets (GSE10780, GSE15852, GSE29044, GSE37751, GSE70905, GSE70947, GSE93601, GSE83591, GSE109169, and GSE139038) from the GEO database (https://www.ncbi.nlm.nih.gov/geo) were used. The datasets were required to meet the following criteria: (1) microarray expression profiles of *Homo sapiens*; (2) inclusion of paired normal or adjacent tissues and cancer tissues; (3) sample size ≥30 for each dataset. A total of 29 and 43 samples from GSE29044 and GSE70905, respectively, were excluded because they did not meet these criteria. Moreover, the overall survival data and clinical outcomes of dataset GSE37751 were used to validate the survival analysis. The sample statistics of the 10 GEO datasets are given in **Supplementary Table S1**.

To identify robust DEGs across all BC datasets, we incorporated both nonparametric (NOISeq) and RRA-ranked parametric (EdgeR) methods in the differential expression analysis. NOISeq calculates M (the log_2_ ratio of the 2 conditions) and D (the value of the difference between conditions) values to capture the probability of differential expression^13^, with a q value cutoff of 0.8 set for each dataset. The distribution of nonparametric DEGs from NOISeq was visualized in a Manhattan plot with the MDplot function. The parametric DEGs were normalized with the R package limma and determined with the EdgeR package in R 4.0.1 software. The cutoff criteria for the DEGs were |log_2_ fold change (FC)| > 1 and *P*-value < 0.05, and the distribution of DEGs from EdgeR was visualized in a volcano plot with the R package ggplot2. The parametric DEGs across the 10 individual datasets were subsequently ranked with the RRA method, which can avoid multiple testing errors^12^, performed with the RobustRankAggreg R package. The robust DEGs were screened by integrating NOISeq and RRA-ranked EdgeR methods in multiple GEO dataset. A network graph of the interactions between the regulated robust DEGs mapping to the PPI network from STRING were plotted with Cytoscape 3.80^15^.

### Single sample gene set enrichment analysis (ssGSEA) and hierarchical clustering analysis

The immunological signature of each TCGA sample of BC was estimated on the basis of pre-defined immune gene sets with the ssGSEA algorithm in the R package GSVA^16^. The immune gene sets indicated the biological functions, chromosomal localization, and physiological regulation of 28 types of immune cells. The bar plot of immune cell proportions was visualized with the ggplot2 package. The high- and low-immune cell infiltration subtypes of the patients were identified through hierarchical clustering analysis based on Euclidean distance. The T-distribution stochastic neighbor embedding (t-SNE) algorithm was used to assess the precision in discriminating immune subtypes.

### Immune cell deconvolution analysis with CIBERSORT and XCELL algorithms

The proportions of 22 types of tumor-infiltrated immune cells were estimated with the CIBERSORT and XCELL algorithms, on the basis of TCGA breast tumor gene expression profiles^17^. The gene expression profiles for 1,045 samples were normalized and deconvoluted into the proportions of the 22 types of immune cells, and the *P*-value cutoff was set to 0.05. The box plots were visualized with the R package ggplot2.

### Correlation analysis between hub gene expression and immune cell infiltration

The proportions of 6 types of immune cells—CD4+ T cells, CD8+ T cells, neutrophils, macrophages, and myeloid dendritic cells (DCs)—were estimated with the TIMER immune deconvolution method to establish potential correlation between hub gene expression and immune cell infiltration in TCGA breast tumor cohort^18^. Pearson’s correlation coefficient was calculated to assess the fitted linear relationship with a significance threshold of |r| > 0.3 and *P* < 0.01. The protein expression of hub genes was verified with the Human Protein Atlas database (HPA, https://www.proteinatlas.org).

### Survival analysis and hub gene-associated prognostic models

The gene expression profiles and corresponding clinical data from the TCGA BC cohort and the microarray dataset GSE37751 were used for survival analysis. The hub-gene based risk signature was subjected to univariate and multivariate Cox regression analysis to build a hub-gene associated prognosis model. The risk score was calculated with the formula described by Guan et al.^19^. The low- and high-risk groups of TCGA patients were divided according to the median value of the hub gene-based risk signature, and survival analysis was performed with the Kaplan-Meier method. The log-rank test was used to test the differences in survival rates between groups. The time-dependent receiver operating curve (ROC) was generated to reflect the predictive ability of the hub gene-based risk signature, and the area under the curve (AUC) for 1-, 3-, and 5-year overall survival was calculated. The Kaplan-Meier, log-rank, ROC curve, and calibration analyses were performed and visualized with the survival, survminer, timeROC, and rms packages. The relationship between the hub-gene-based risk signature and clinical factors in discrete immune cell infiltration groups was analyzed with Pearson’s correlation. *P*-values < 0.05 were considered statistically significant. A nomogram was established to predict the hub-gene-based risk signature, and could make a comparison with clinical factors. ROC and calibration curve analysis were used to determine the robustness. The association between survival and the hub-gene-based risk signature was validated with an independent dataset, GSE37751.

## Results

### Differences in the proportions of immune cells in 2 subtypes of BC

**Figure 1:**
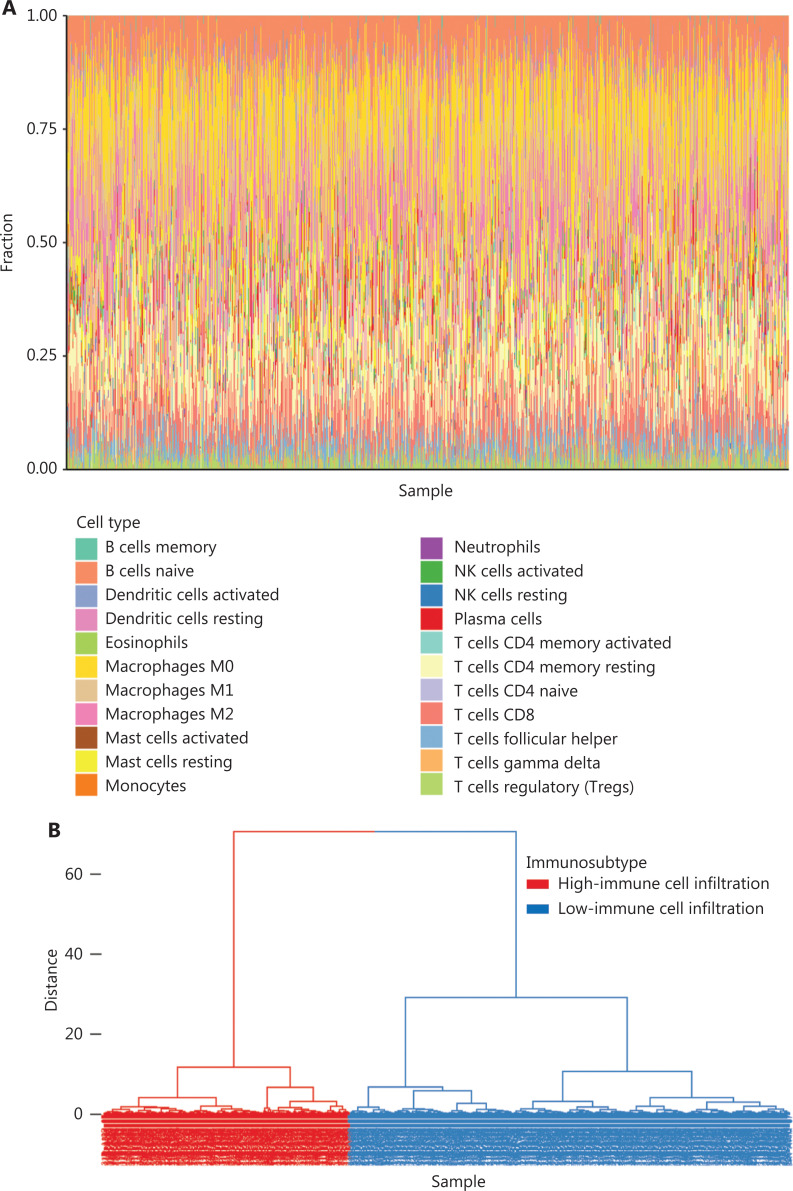

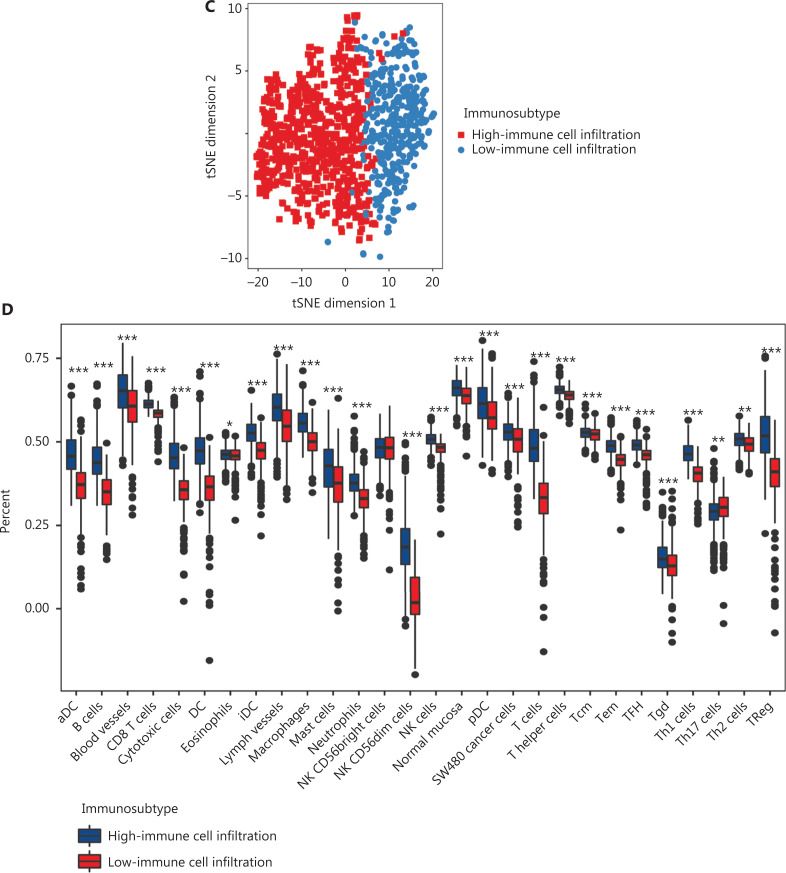
Hierarchical clustering of patients with BC in TCGA cohort. (A) Distribution of 22 types of immune cells, determined with CIBERSORT. (B) Hierarchical clustering division of BC patients into high- and low-immune cell infiltration by ssGSEA. (C) Validation of immunophenotype *via* t-SNE. (D) Immune cell infiltration levels between subtypes. Student’s t test: *P < 0.05, **P < 0.01, ***P < 0.001.

**Figure 2:**
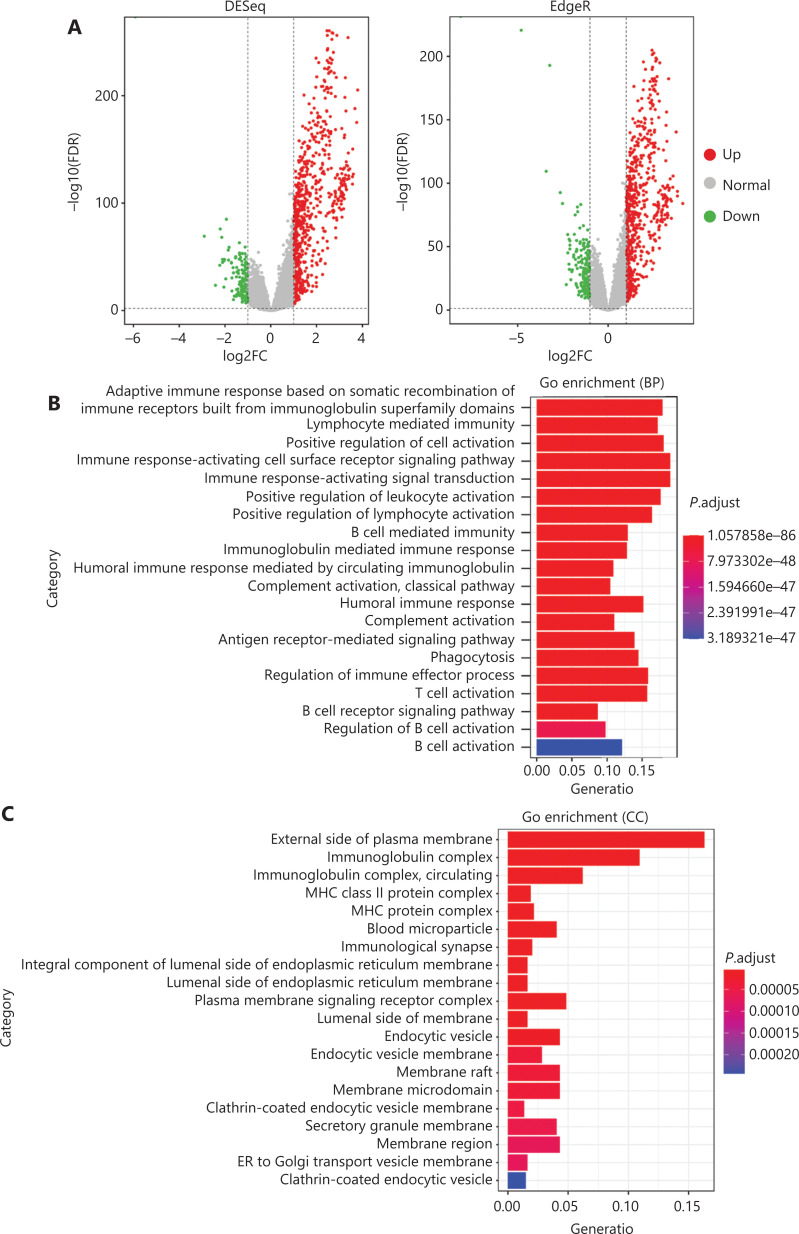

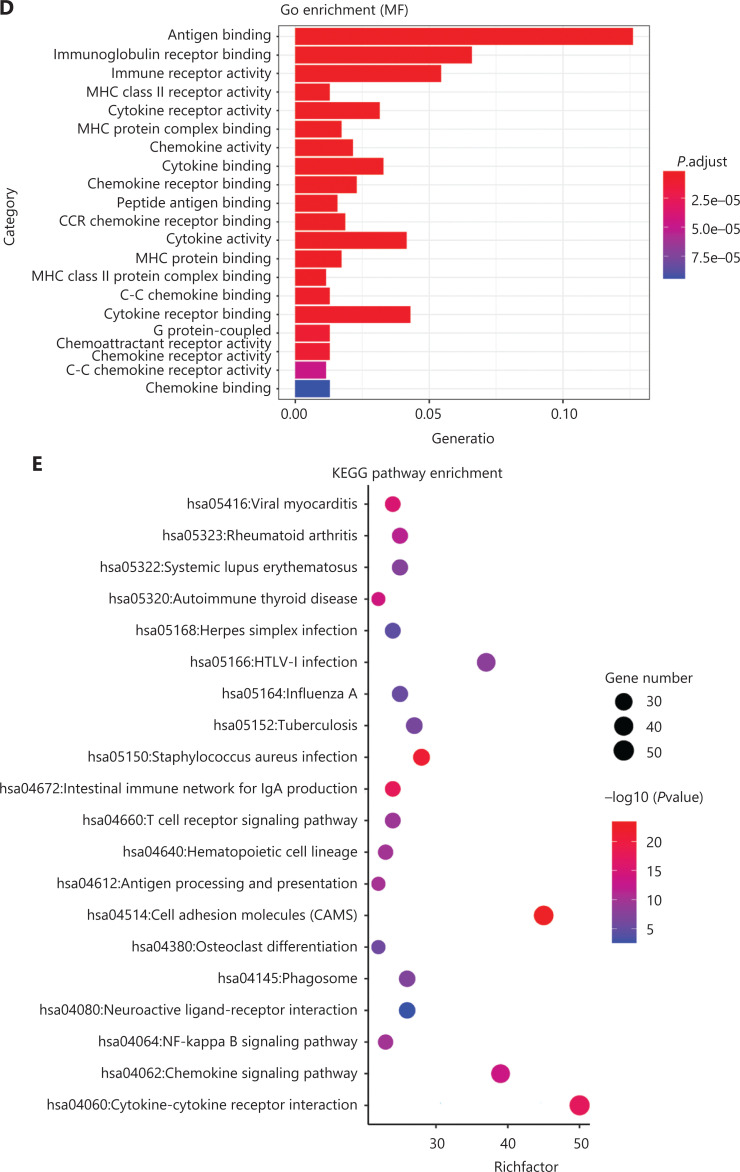
Functional enrichment analyses of BC samples from TCGA cohort. (A) Volcano plots of DEG distribution, on the basis of 2 R packages, DESeq and EdgeR. Red and green dots represent up- and down-regulated genes, respectively. (B–D) GO enrichment analyses of DEGs in (B) biological process (BP), (C) cellular component (CC), and (D) molecular function (MF) terms. (E) KEGG enrichment analysis of DEGs.

To comprehensively evaluate the immunological characteristics in BC, we analyzed 1,045 tumor samples from TCGA cohort with the CIBERSORT algorithm, and generated a difference heatmap of 22 types of immune cells (**Figure 1A**). On the basis of the ssGSEA scores and hierarchical clustering algorithm, we clustered the samples into high- and low-immune cell infiltration groups (**Figure 1B**), and confirmed the immune level clustering by using the t-SNE algorithm, which also revealed the same categories (**Figure 1C**). Next, we found that the degree of infiltration of most the 28 types of immunity-associated cells significantly differed among categories (**Figure 1D**). To investigate the molecular characteristics underlying the different immunophenotypes, we calculated the DEGs with the R packages DESeq and EdgeR, and identified up-regulated and down-regulated genes (**Figure 2A**). We performed GO term and KEGG pathway enrichment analyses by using the R packages to assess the biological functions of the DEGs. The biological process (BP) terms were markedly enriched in immune responses (**Figure 2B**), whereas the cellular component (CC) and molecular function (MF) terms were enriched in cellular functions on the outer plasma membrane (*P* = 1.86E-73) and antigen binding (*P* = 5.86E-80), respectively (**Figure 2C and 2D**). Additionally, KEGG pathway analysis revealed that the DEGs were significantly enriched in cytokine-cytokine receptor interaction (*P* = 6.19E-18), cell adhesion molecules (*P* = 4.20E-24), and chemokine signaling pathway (*P* = 3.28E-14) (**Figure 2E**). These results suggested that these DEGs have crucial functions in the immunological characteristics of BC.

### Robust DEGs in multiple GEO datasets for BC

**Figure 3:**
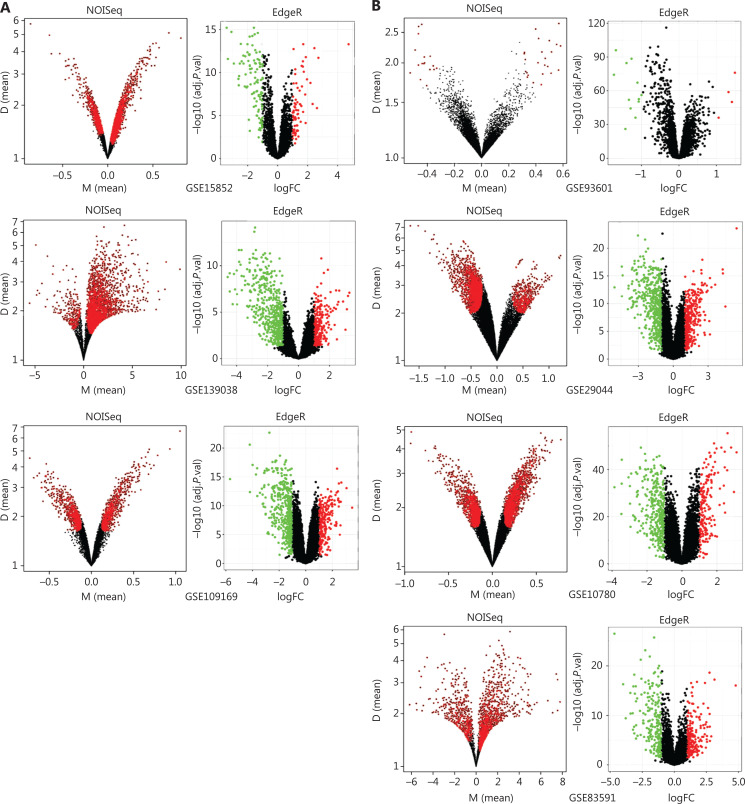

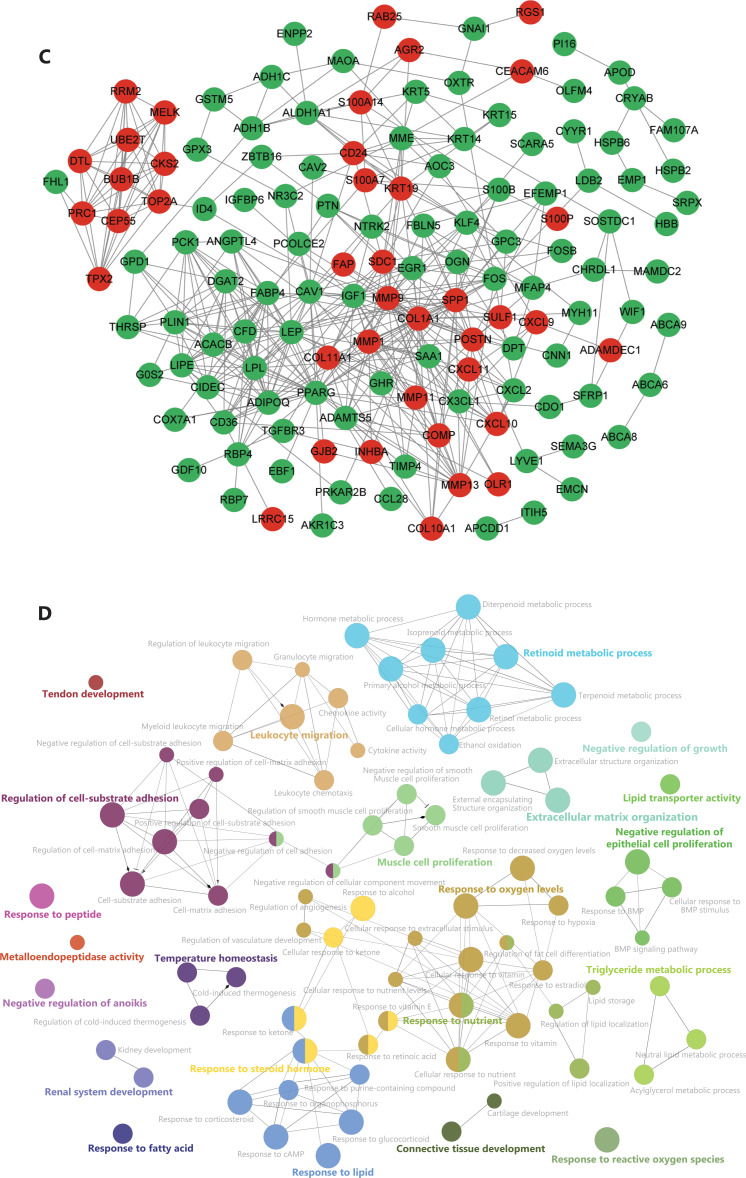
Identification and enrichment analysis of robust DEGs. (A–B) Volcano plots of the DEG distribution in BC from GEO datasets: (A) tumors *vs.* normal tissue datasets (GSE15852, GSE139038, and GSE109169) and (B) tumors *vs.* adjacent tissue datasets (GSE93601, GSE29044, GSE10780, and GSE83591). Red and green dots represent up- and down-regulated genes, respectively, in EdgeR in the R package. (C) PPI network of the robust DEGs. Red and green nodes represent up- and down-regulated genes, respectively. (D) Enrichment analysis of robust DEGs with Clue GO in Cytoscape.

To explore the functional DEGs, we performed integrated bioinformatics analyses of the GEO datasets (**Supplementary Figure S1**). Ten microarray datasets (GSE10780, GSE15852, GSE29044, GSE93601, GSE83591, GSE109169, GSE139038, GSE37751, GSE70905, and GSE70947) were divided into 2 groups including 7 test datasets, which were analyzed with both nonparametric (NOISeq) and RRA-ranked parametric (EdgeR) methods to obtain the robust DEGs; 3 datasets were used for additional validation (**Supplementary Table S1**). The thresholds used in NOISeq and EdgeR were consistent with the standard^20,21^. The upregulated and downregulated genes are shown in volcano plots (**Figure 3A, 3B and Supplementary Figure S2A**). Moreover, 163 robust DEGs, including 43 upregulated and 120 downregulated genes with adjusted *P*-value < 0.05, were screened by intersecting the above nonparametric NOISeq and parametric EdgeR methods, thus overcoming the heterogeneity in different datasets. (**Supplementary Table S2**). We then imported the robust DEGs into the STRING database to construct a PPI network, which was visualized in Cytoscape (**Figure 3C**), and the robust DEGs were enriched in multiple biological processes and functions (**Figure 3D**).

### Identification of sub-networks and hub genes

**Figure 4:**
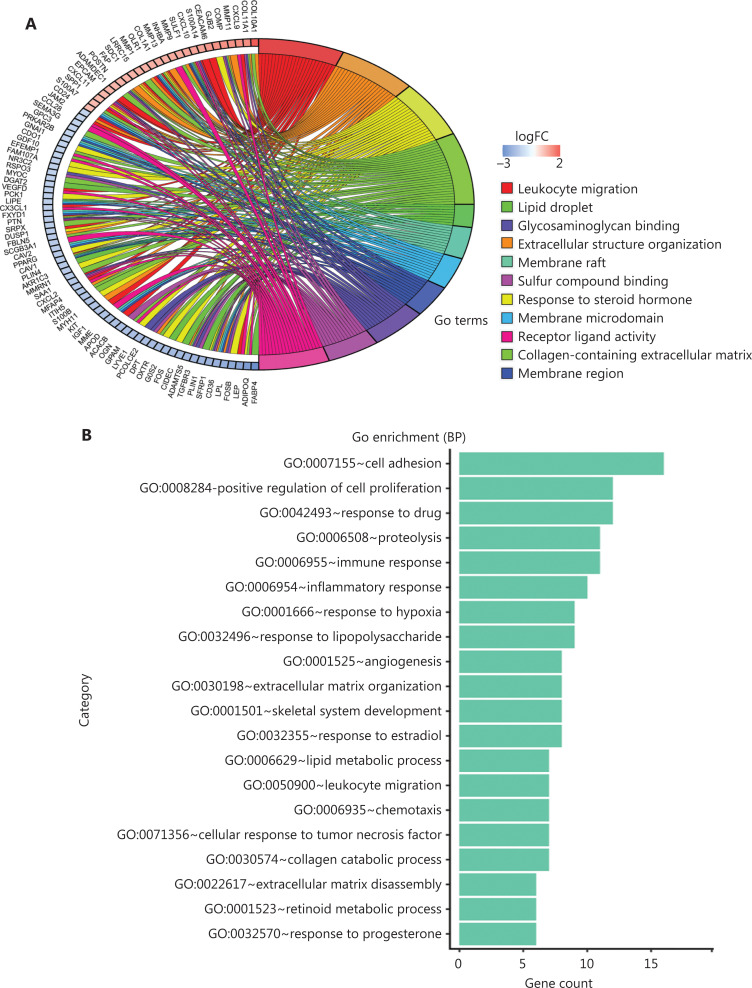

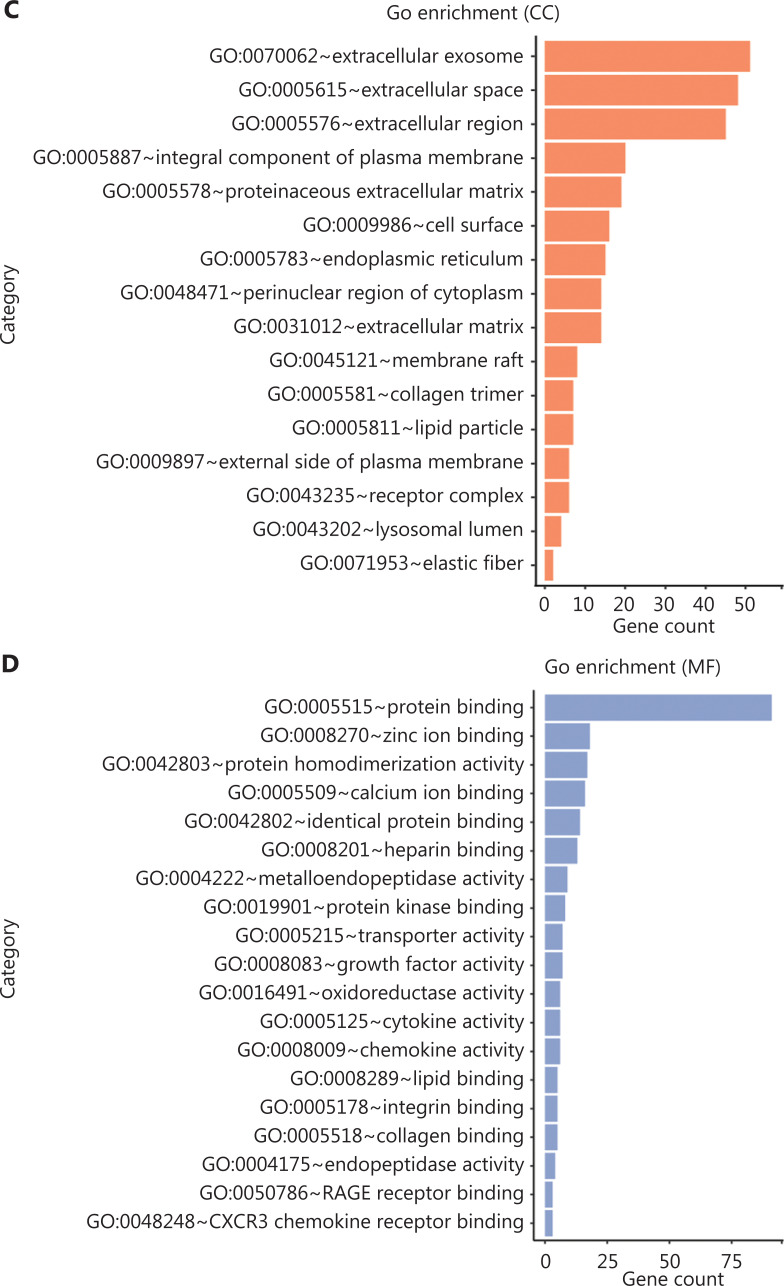

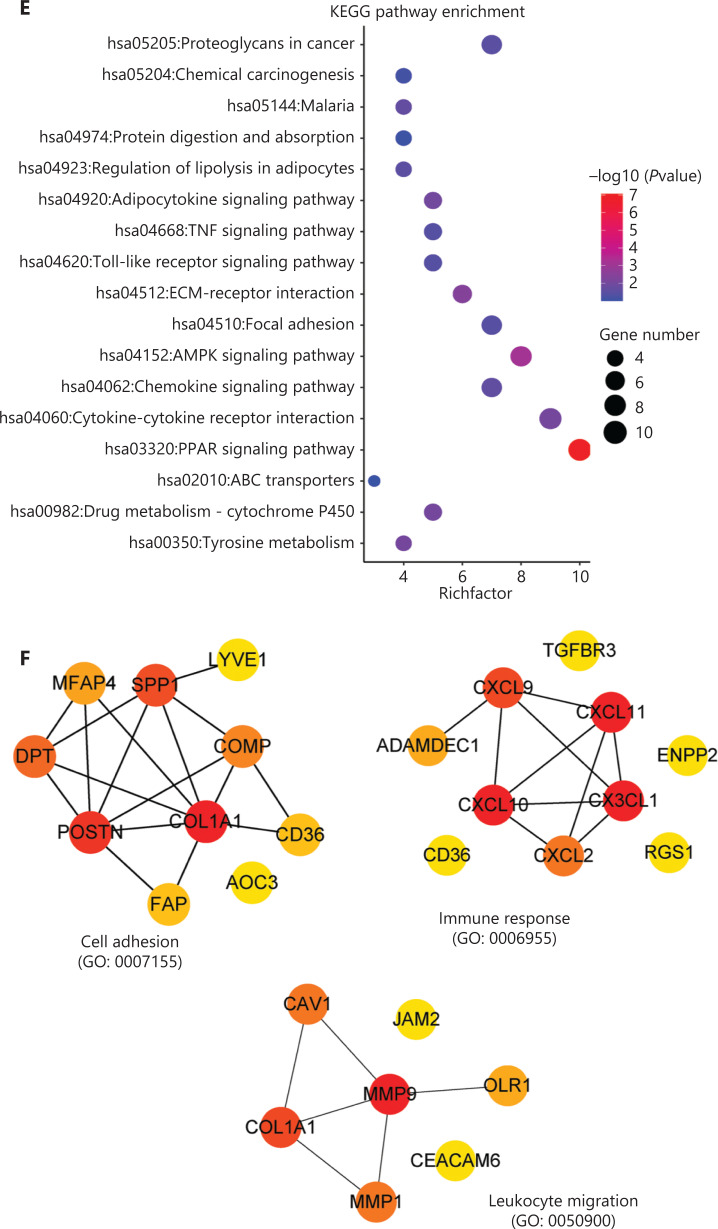
Functional analysis of robust DEGs and identification of hub genes. (A) GO term enrichment analysis of overlapping robust DEGs. (B–D) GO enrichment analyses of robust DEGs in 3 terms: (B) biological process (BP), (C) cellular component (CC), and (D) molecular function (MF). (E) KEGG pathway enrichment analysis of robust DEGs. (F) Representative sub-network of immune-associated robust DEGs identified with CytoHubba in Cytoscape.

To further investigate the biological functions of these robust DEGs, we performed GO and KEGG pathway enrichment analyses (**Supplementary Table S3**). Analysis of overlapping DEGs revealed that most of these robust DEGs appeared in multiple GO terms and KEGG pathways (**Figure 4A and Supplementary Figure S2B**). The significant BP terms included cell adhesion (*P* = 2.51E-05), positive regulation of cell proliferation (*P* = 4.23E-03), response to drug (*P* = 1.30E-04), proteolysis (*P* = 1.85E-02), immune response (*P* = 6.03E-03), and inflammatory response (*P* = 9.03E-03) (**Figure 4B**); the enriched CC terms were mainly extracellular exosome (*P* = 2.31E-07), extracellular space (*P* = 3.34E-17), and extracellular region (*P* = 3.01E-12) (**Figure 4C**); the most enriched MF term was protein binding (*P* = 1.54E-02) (**Figure 4D**). Additionally, KEGG pathway enrichment analysis revealed that the robust DEGs were significantly enriched in PPAR signaling (*P* = 8.44E-08), cytokine-cytokine receptor interaction (*P* = 8.68E-03), chemokine signaling (*P* = 2.44E-02), and AMPK signaling (*P* = 6.64E-04) (**Figure 4E**). The above functional enrichment analysis implicated multiple immune-associated biological processes and pathways involved in BC.

To identify the immune-associated hub genes, we focused on genes enriched in the BP terms of cell adhesion, immune response, leukocyte migration, cell chemotaxis, and inflammatory response, and immune-associated KEGG pathways including cytokine-cytokine receptor interaction, and chemokine signaling pathway (**Figure 4B and 4E**). We imported the robust DEGs into CytoHubba in Cytoscape to build the sub-network, then evaluated the degree of confidence of genes by using MCC topological analysis scoring algorithms to identify the key genes in the sub-network (**Figure 4F, Supplementary Figure S2C and S2D**). The results indicated 10 top-scored hub genes with high confidence: CXCL10, CXCL9, CXCL11, SPP1, POSTN, MMP9, DPT, COL1A1, ADAMDEC1, and RGS1. These 10 genes were considered to be key driver genes participating in immune-associated BC progression (**Figure 4F and Supplementary Table S4**).

### Correlation between hub genes and the abundance of tumor-infiltrating immune cells

**Figure 5:**
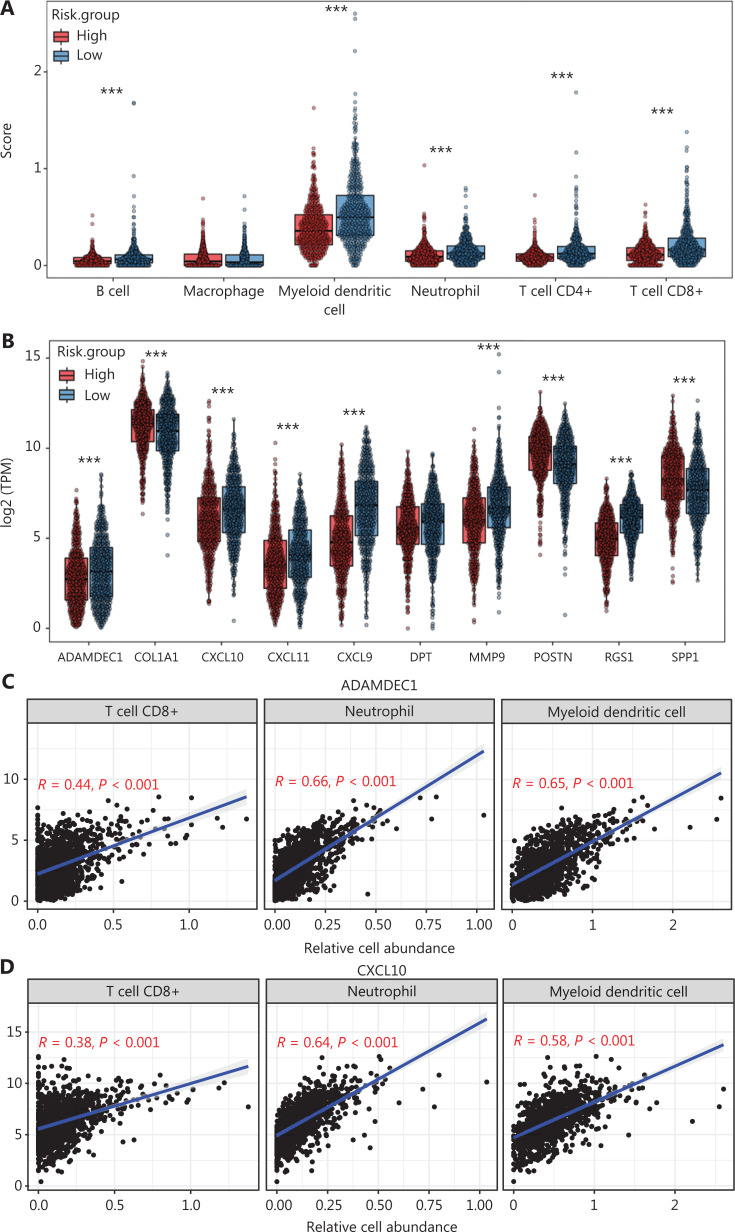

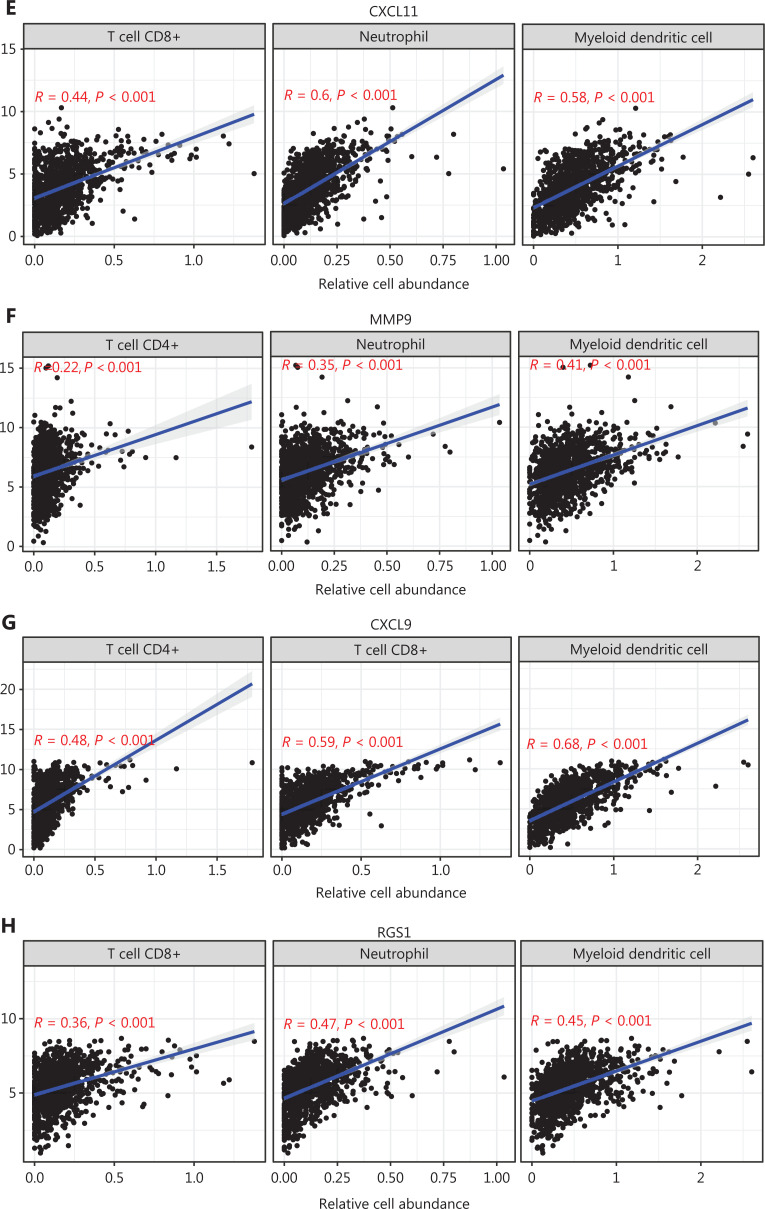

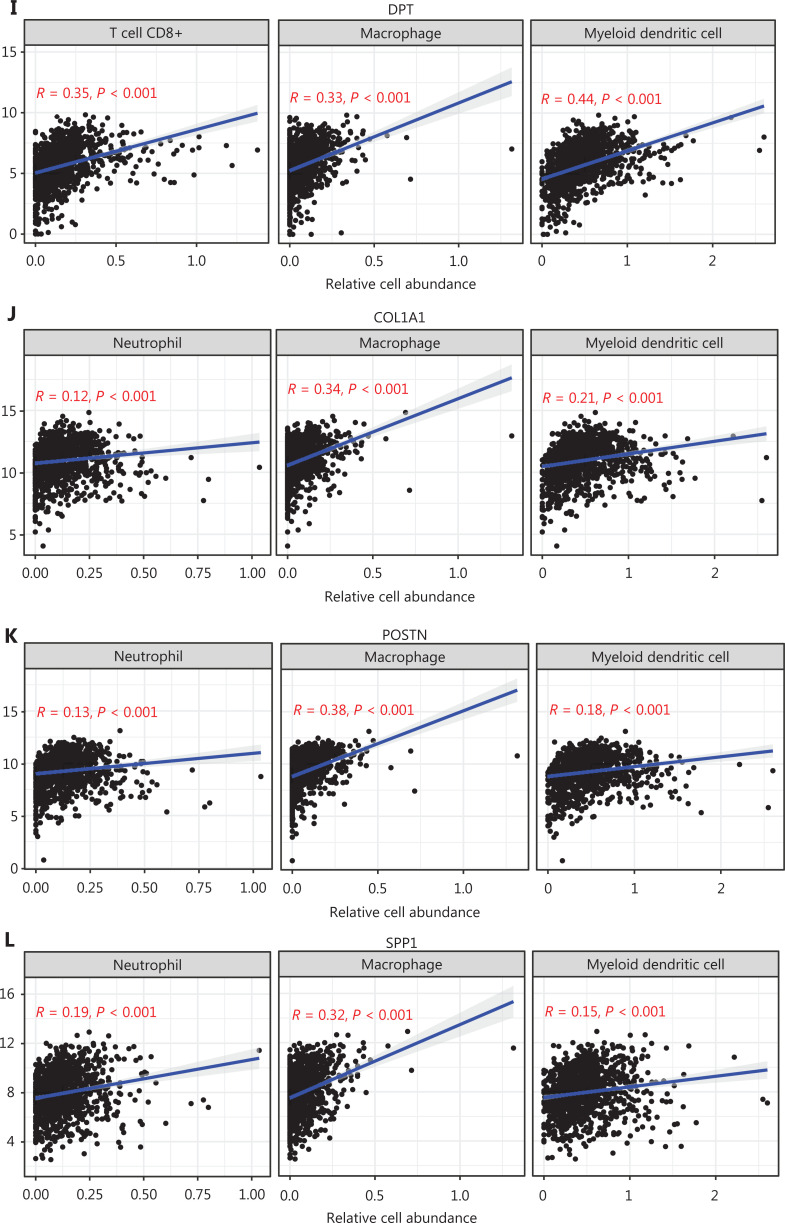
Correlation analysis between hub genes and immune cell infiltration in BC samples from TCGA cohort. (A) Differences in the proportions of immune cells, with a comparison between high- and low-risk groups. (B) Different levels of the 10 hub genes between high- and low-risk groups. (C–L) Correlations between the 10 hub genes and the abundance of the immune cells, deconvoluted with TIMER. Correlation analysis of (C) ADAMDEC1, (D) CXCL10, (E) CXCL11 and CD8+ T cells, neutrophils, and myeloid DCs; (F) MMP9 and CD4+ T cells, neutrophils, and myeloid DCs; (G) CXCL9 and CD4+ T cells, CD8+ T cells, and myeloid DCs; (H) RGS1 and CD8+ T cells, neutrophils, and myeloid DCs; (I) DPT and CD8+ T cells, macrophages, and myeloid DCs; (J) COL1A1; (K) POSTN; and (L) SPP1 and neutrophils, macrophages, and myeloid DCs. (A–B) Student’s t test: ***P < 0.001.

We generated the difference heatmaps of 22 types of immune cells in 1,045 BC samples from TCGA cohort by using the immune deconvolution methods CIBERSORT and XCELL, (**Figure 1A, Supplementary Figure S3A and Supplementary Table S5**). B cells, myeloid DCs, neutrophils, CD4+ T cells, and CD8+ T cells were exhibited significantly differential expression in high- and low-risk groups, as visualized in a box plot (**Figure 5A**). Additionally, we found that the expression levels of CXCL10, CXCL9, CXCL11, SPP1, POSTN, MMP9, COL1A1, ADAMDEC1, and RGS1, but not DPT, significantly differed between the high- and low risk groups (**Figure 5B**). We therefore further explored the significance of correlations between the hub genes and the deconvoluted immune cell abundance by using TIMER. The ratios of neutrophils and myeloid DCs positively correlated with the expression of ADAMDEC1, CXCL10, CXCL11, and MMP9 (**Figure 5C–5F and Supplementary Figure S3B–S3E**), whereas the ratios of CD4+ T cells, CD8+ T cells, and myeloid DCs correlated with the expression of CXCL9 (**Figure 5G and Supplementary Figure S3F**). Myeloid DCs and CD8+ T cells also significantly correlated with the expression of RGS1 and DPT (**Figure 5H, 5I, Supplementary Figure S3G and S3H**). Moreover, the levels of RGS1 and DPT correlated with higher abundance of neutrophils and macrophages, respectively (**Figure 5H, 5I, Supplementary Figure S3G and S3H**), and the expression of COL1A1, POSTN, and SPP1 correlated with the abundance of macrophages (**Figure 5J–5L and Supplementary Figure S3I–S3K**). Furthermore, we verified the protein expression levels of these hub genes in HPA^22^ and found that the perturbation pattern of each identified hub gene was consistent with that in the HPA BC database (**Supplementary Figure S4**). These findings suggested that these hub genes are associated with modulation of functionally important immune cell populations infiltrated into tumors, thus considerably contributing to the modulation of tumor immunity in BC.

### Prognostic value of hub genes in survival of patients with BC

**Figure 6:**
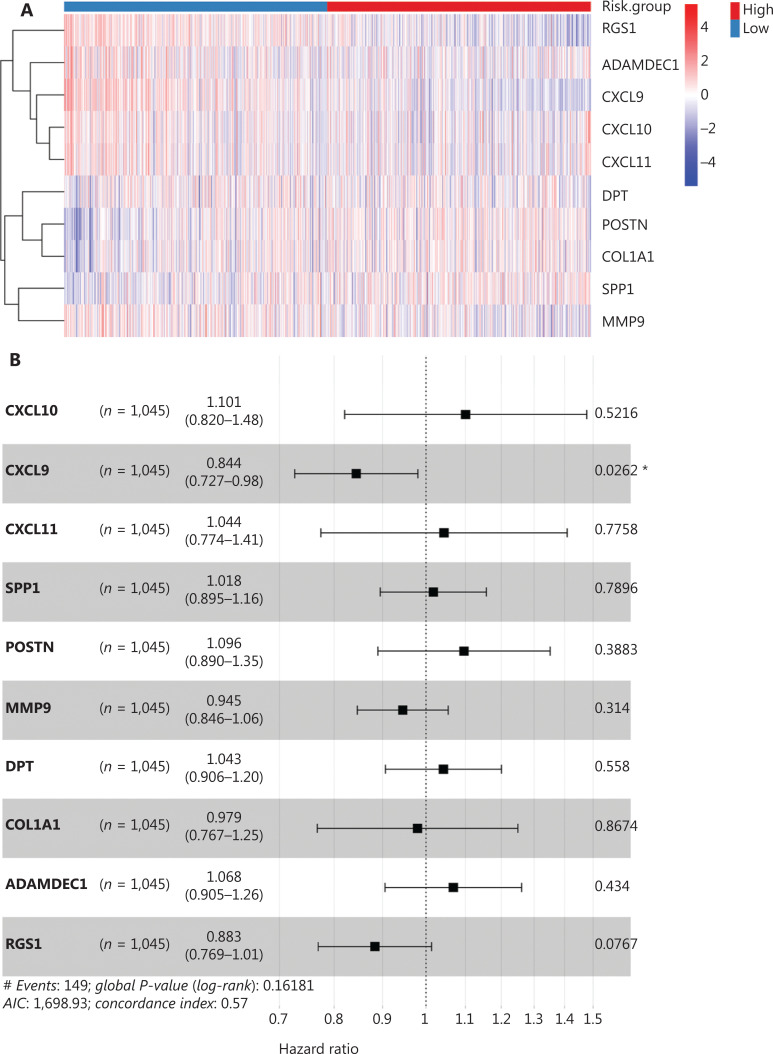

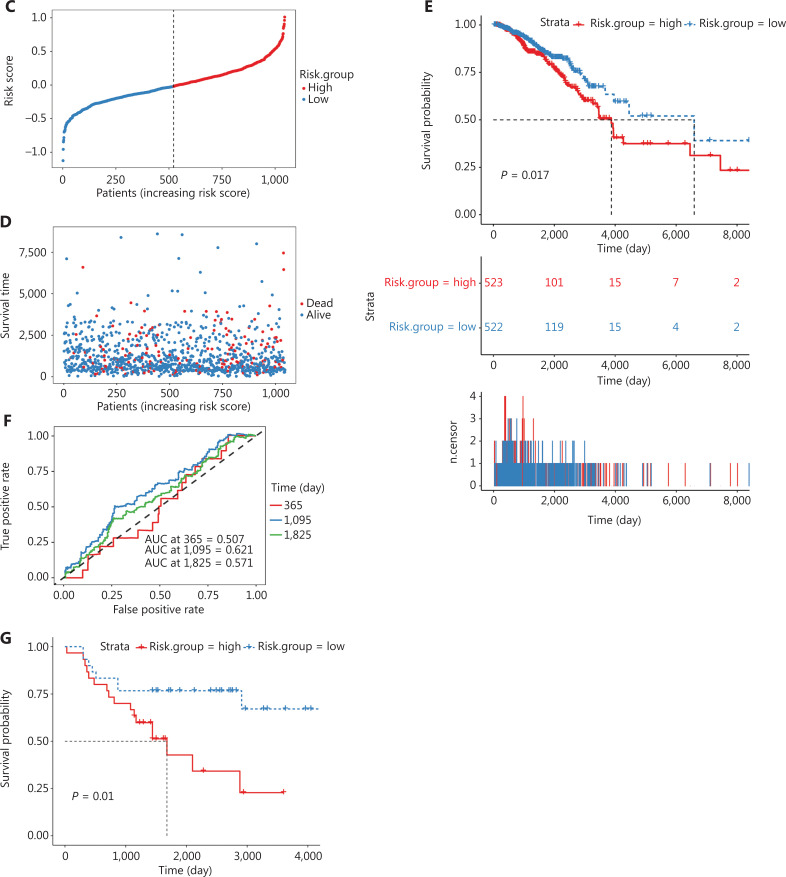
Construction and validation of the hub-gene-based risk signature in BC survival. (A) Heatmaps of the expression of the 10 hub genes in high- and low-risk groups in TCGA dataset. (B) Forest plots of the hazard ratios of the hub genes, by univariable Cox proportional hazards regression analysis. (C–D) Distribution of risk score, survival time, and survival status in TCGA cohort. (E) Kaplan–Meier survival analysis. (F) ROC curve based on the hub-gene-based risk signature of TCGA cohort. (G) Survival curves of patients with BC in the validation dataset GSE37751 (*n* = 60). Log-rank test: *P < 0.05.

To assess the prognostic value of the hub genes, we preformed survival analysis to assess the overall survival of 1,045 BC patients from TCGA. According to risk scores calculated from the 10 hub gene expression profiles, we divided patients into high- and low-risk groups (**Figure 6A**). Six of the 10 hub genes were risk-favorable, with a hazard ratio greater than 1.0, whereas CXCL9 and RGS1 appeared to be risk-unfavorable (**Figure 6B**). The 2 groups were further distinguished by calculation of the risk score for each patient according to the median risk score (**Figure 6C**). With increasing risk scores, the patient survival rate decreased, and the incidence of death increased (**Figure 6D**). Additionally, the overall survival rates of the patients in the high-risk group were significantly poorer in the survival strata (*P* = 0.017, **Figure 6E**). The time-dependent ROC curve analysis supported the predictive robustness and accuracy of the hub gene-based risk signature (**Figure 6F**). Furthermore, similar results were achieved in the validation dataset from GEO (GSE37751, *n* = 60) (**Figure 6G and Supplementary Figure 5E**): the high-risk group exhibited a lower overall survival rate (*P* = 0.01). These findings indicated that the 10 identified hub genes, when used together, have considerable prognostic value for BC.

**Figure 7:**
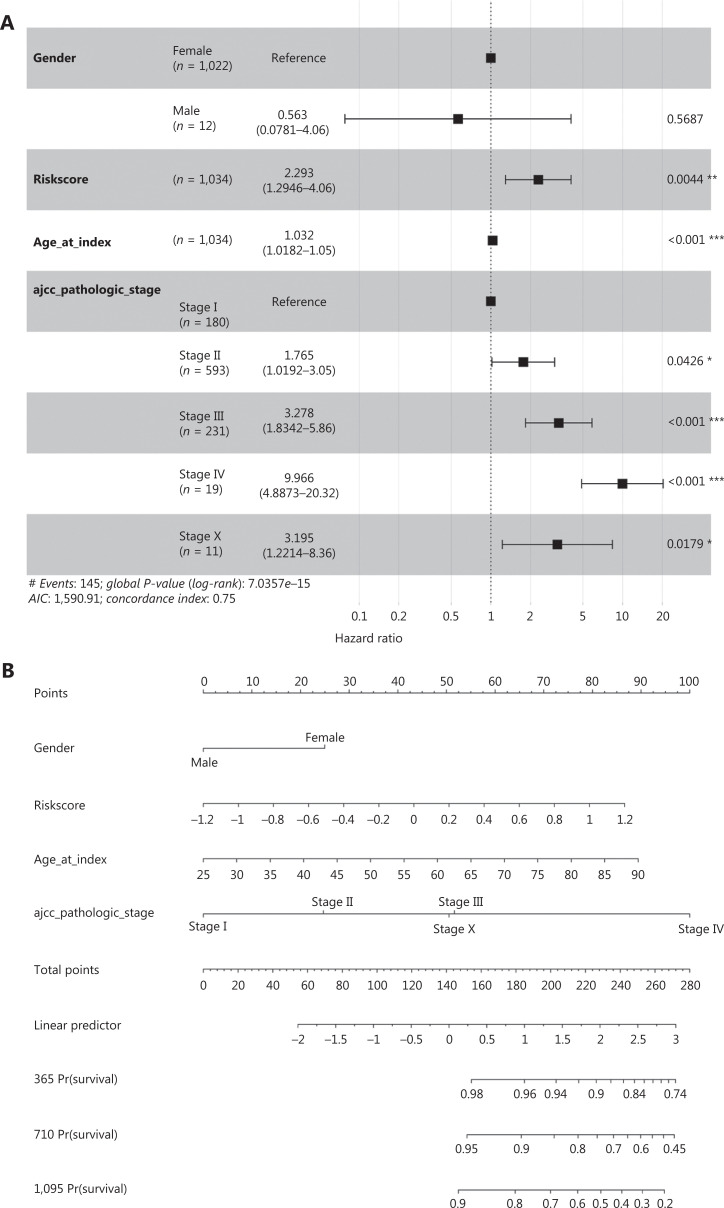

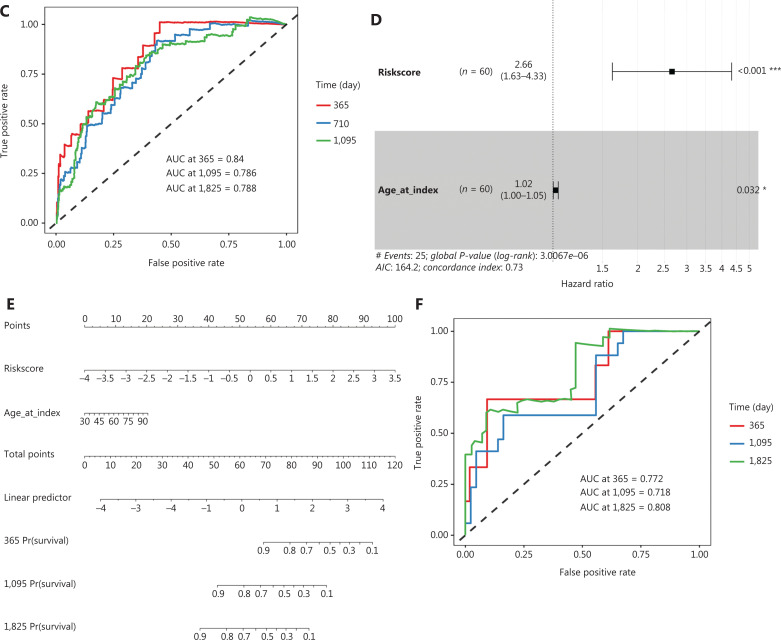
Validation of the hub gene-based risk signature by multivariate Cox analysis and nomogram analysis. (A) Forest plot of the hazard ratios of the gene-based risk signature, by multivariate Cox proportional hazards regression analysis in TCGA cohort. Log-rank test: *P < 0.05, **P < 0.01, ***P < 0.001. (B) Nomogram for clinical characteristics and the gene-based risk signature in TCGA cohort. (C) ROC curves and AUC of the predictions for 1-, 3-, and 5-year of the nomogram for TCGA cohort. (D) Forest plot of hazard ratios of the gene-based risk signature by multivariate Cox proportional hazards regression analysis in dataset GSE37751. (E) Nomogram for clinical characteristics and the hub-gene-based risk signature in dataset GSE37751. (F) ROC curve and AUC of the predictions for 1-, 3-, and 5-year of the nomogram in dataset GSE37751. Log-rank test: *P < 0.05, ***P < 0.001.

We therefore compared the prognostic value of the 10-hub-gene-based risk score with that of two common risk factors—age and American Joint Committee on Cancer (AJCC) stage—and found that the 10-hub-gene-based risk score had significantly greater prognostication power (*P* = 0.0044) (**Figure 7A, 7B and Supplementary Table S6**). The ROC curve indicated the predicted overall survival with the 10-hub-gene-based risk score was robust among all survival strata (1-, 3- and 5-year survival; **Figure 7C**). The robust prognostic value of the 10-hub-gene-based risk score was confirmed with the validation dataset GSE37751 (**Figure 7D–7F**). These data indicated that the identified hub genes have substantial value in BC prognostication.

## Discussion

Leveraging biological and technical heterogeneity across multiple independent datasets is increasingly recognized to aid in identifying robust and reproducible gene signatures^23–25^. We integrated multiple GEO datasets in this study by using both nonparametric and parametric methods, and identified 163 robust DEGs. By further integration with data from TGCA and GEO, we found that these robust DEGs were enriched in immune-associated processes and pathways. Subsequently, deconvolution of the expression profiles of these robust DEGs with CIBERTSORT and TIMER indicated that the DEG expression was significantly associated with immune-infiltration in BC. Together, these identified hub genes have considerable prognostic value. These findings support that the integrated analyses of cross-library datasets can both overcome dataset size limitations and avoid the issue of data heterogeneity, thus revealing reasonably unbiased causal associations regarding cancer biomarker discovery through RNA-Seq.

We identified 10 immune-associated hub genes—CXCL10, CXCL9, CXCL11, SPP1, POSTN, MMP9, DPT, COL1A1, ADAMDEC1, and RGS1—that may potentially serve as diagnostic and prognostic markers of BC. The survival analysis and multivariate Cox analysis of patients with BC revealed that these hub genes together may serve as an independent risk factor for clinical prognosis. By using immune deconvolution analysis to determine the abundance of immune cells, we found that the expression of hub genes was closely associated with the infiltration of CD8+ T cells, CD4+ T cells, neutrophils, macrophages, and myeloid DCs, which are characteristic of BC progression. However, further investigation of the biological functions and underlying mechanisms is needed.

Among the 10 hub genes identified, CXCL9, CXCL10, and CXCL11 encode chemokines that participate in the modulation of immune cell infiltration in BC, in agreement with previous reports^26,27^; SPP1, POSTN, and COL1A1, which are elevated in patients with high-risk BC, are positively correlated with macrophage infiltration; and MMP9 is functionally correlated with infiltrating neutrophils and DCs. Interestingly, RGS1 was positively correlated with the abundance of neutrophils, myeloid DCs, and CD8+ T cells in tumors, and RGS1 expression was markedly down-regulated in patients with high tumor immune infiltration. Notably, DPT and ADAMEC1, which have scarcely been reported in BC, were found to be associated with CD8+ T cell, neutrophil, and myeloid DC infiltration. Thus, these hub genes might participate, alone or jointly, in the modulation of immune cell content in tumors. The finding that our 10-hub-gene-based risk score had significantly greater prognostication ability than the traditional risk score based on age and AJCC stages indicated that these hub genes together may warrant further investigation to elucidate their roles in the creation and maintenance of inflammatory microenvironments such as those in BC.

The bioinformatics databases that are currently publicly available generally lack multi-omic data from other omics resources, such as copy number variants, DNA methylation profiles, and mRNA and protein post-translational modifications^28,29^, thus rendering the results of pipeline analysis potentially problematic. Therefore, assessing the roles of hub genes, such as those presented here, at multi-omic levels^30^ should further reveal the pathological mechanisms underlying the actions of the 10 hub genes as a group in the modulation of immune cell content and functions in BC.

## Conclusions

In summary, by using integrated bioinformatics analyses of multiple datasets of gene expression profiles in BC in clinical settings, we identified 10 robust hub genes—CXCL10, CXCL9, CXCL11, SPP1, POSTN, MMP9, DPT, COL1A1, ADAMDEC1, and RGS1—that together may serve as a risk factor for BC diagnosis and prognostication.
