# Supplementary material for: Hub genes associated with immune cell infiltration in breast cancer, identified through bioinformatic analyses of multiple datasets
Source: Cancer Biol Med. 2022 Jul 13;19(9):1352–74. doi: 10.20892/j.issn.2095-3941.2021.0586 (PMC9500228; doi:10.20892/j.issn.2095-3941.2021.0586)
Supplement: Supplementary file 1 [file cbm-19-1352-s001.pdf]

Supplementary materials

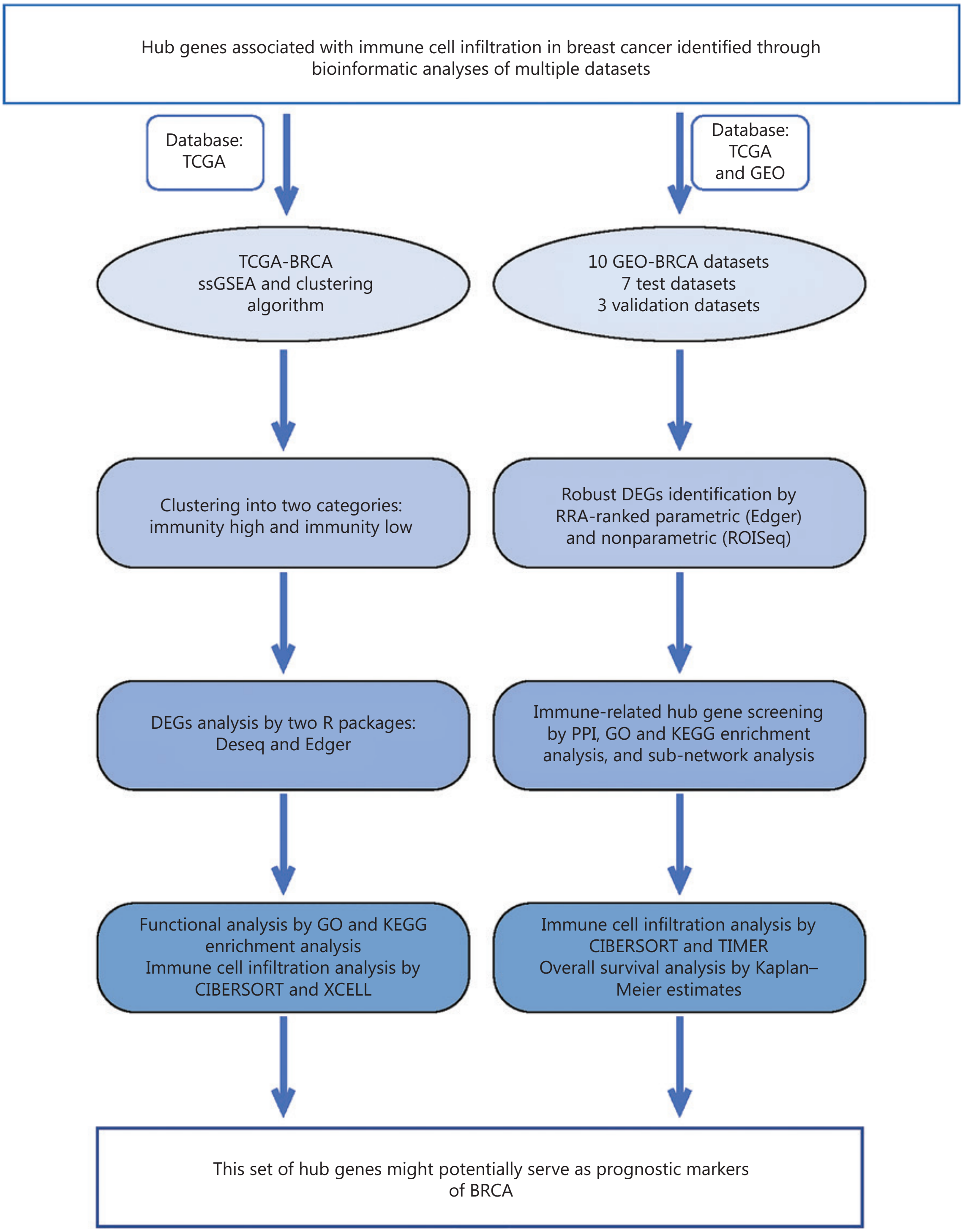

Figure S1 Schematic illustration of the integrated bioinformatics analysis of multiple datasets.

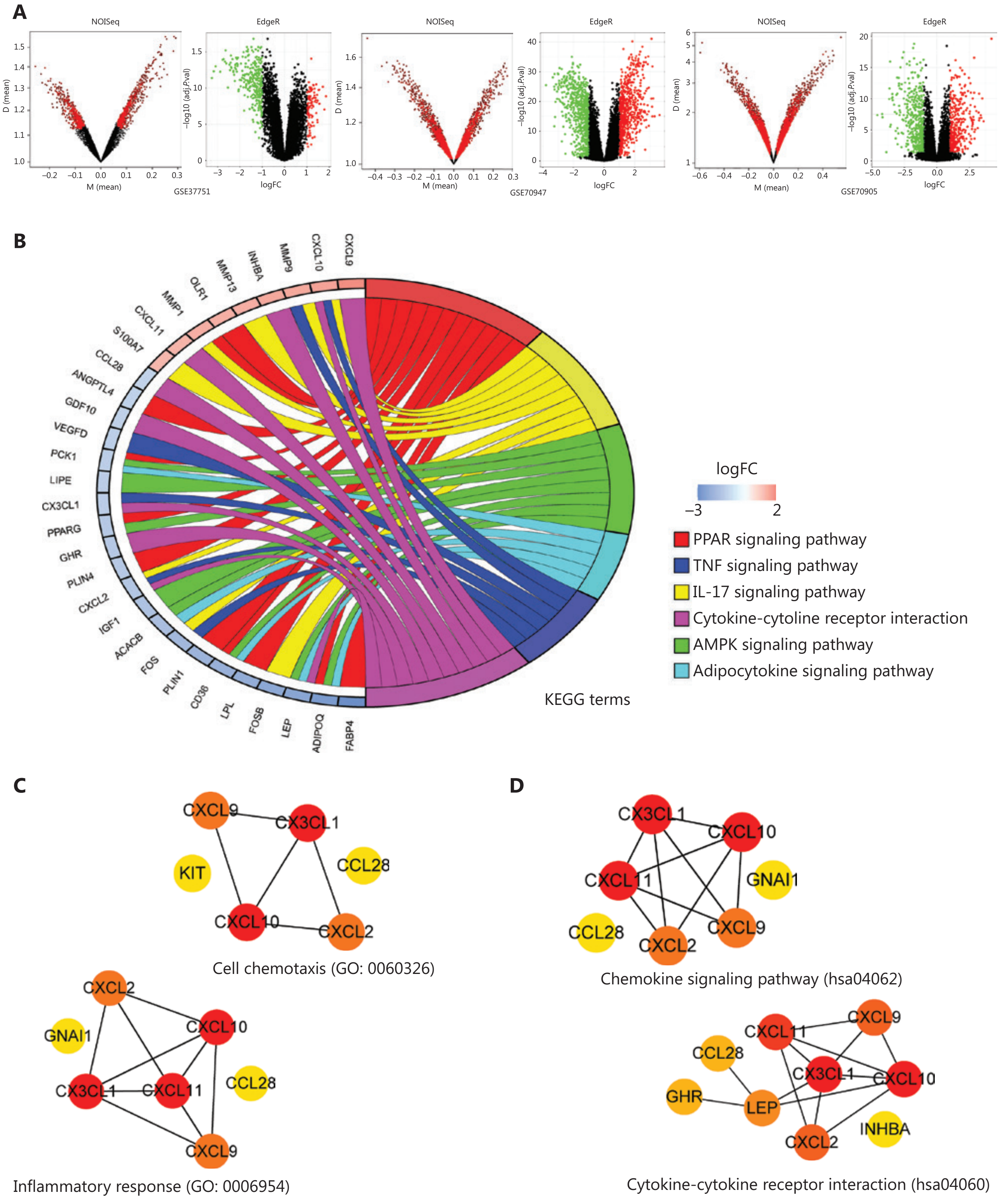

Figure S2 Identification, KEGG enrichment, and subnetwork analyses of the robust DEGs. (A) Volcano plots of the distribution of DEGs in BC from GEO datasets (GSE37751, GSE70947, and GSE70905). Red and green dots represent up- and down-regulated genes, respectively, in EdgeR of the R package. (B) KEGG pathway enrichment analysis of overlapping robust DEGs. (C–D) Representative sub-networks of immune-associated robust DEGs, identified by CytoHubba in Cytoscape.

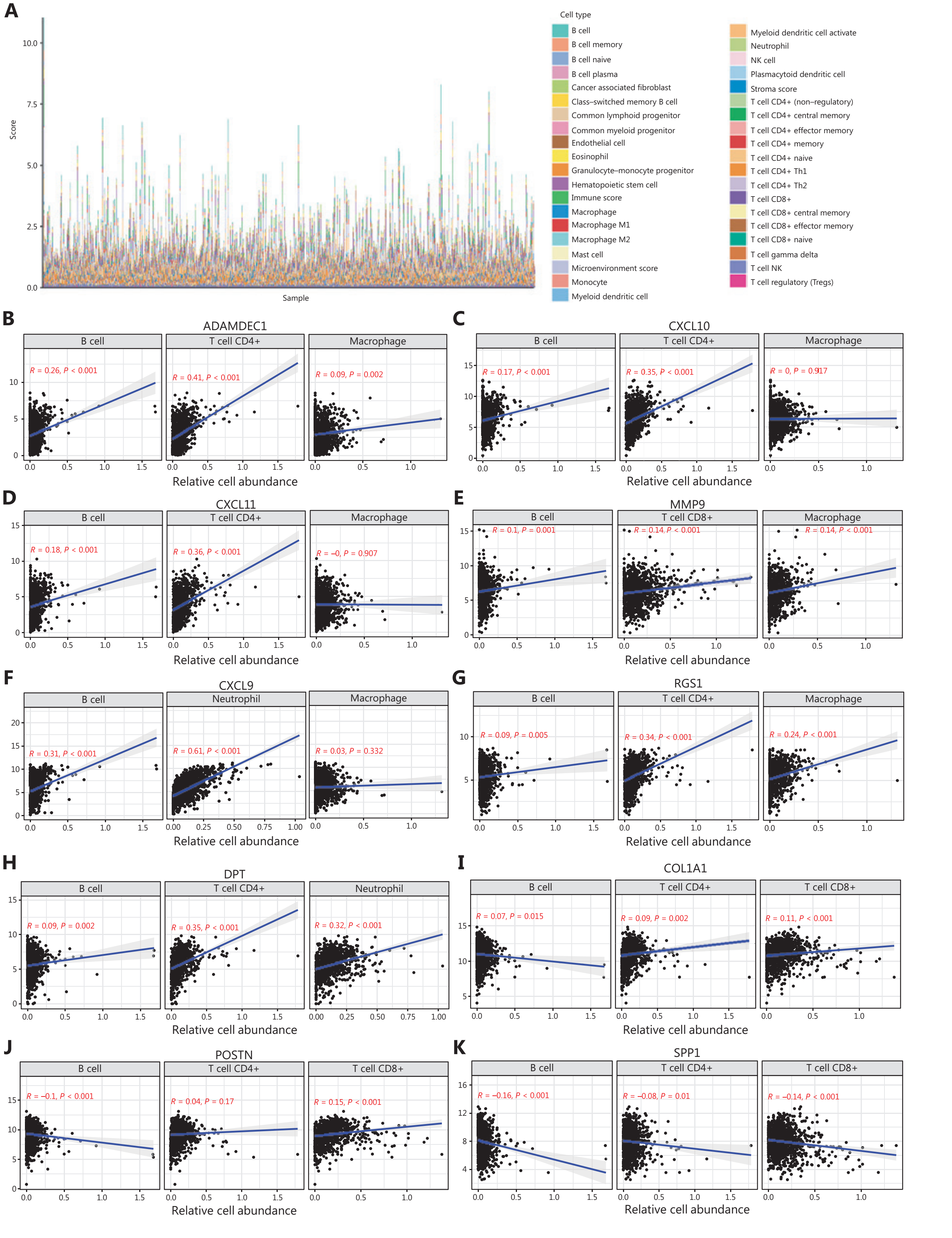

Figure S3 Immune correlation analysis of hub genes. (A) Distribution of 22 types of immune cells, determined by the XCELL immune deconvolution method. (B) ADAMDEC1, (C) CXCL10, (D) CXCL11, (E) MMP9, (F) CXCL9, (G) RGS1, (H) DPT, (I) COL1A1, (J) POSTN, and (K) SPP1 were estimated *via* the TIMER immune deconvolution method.

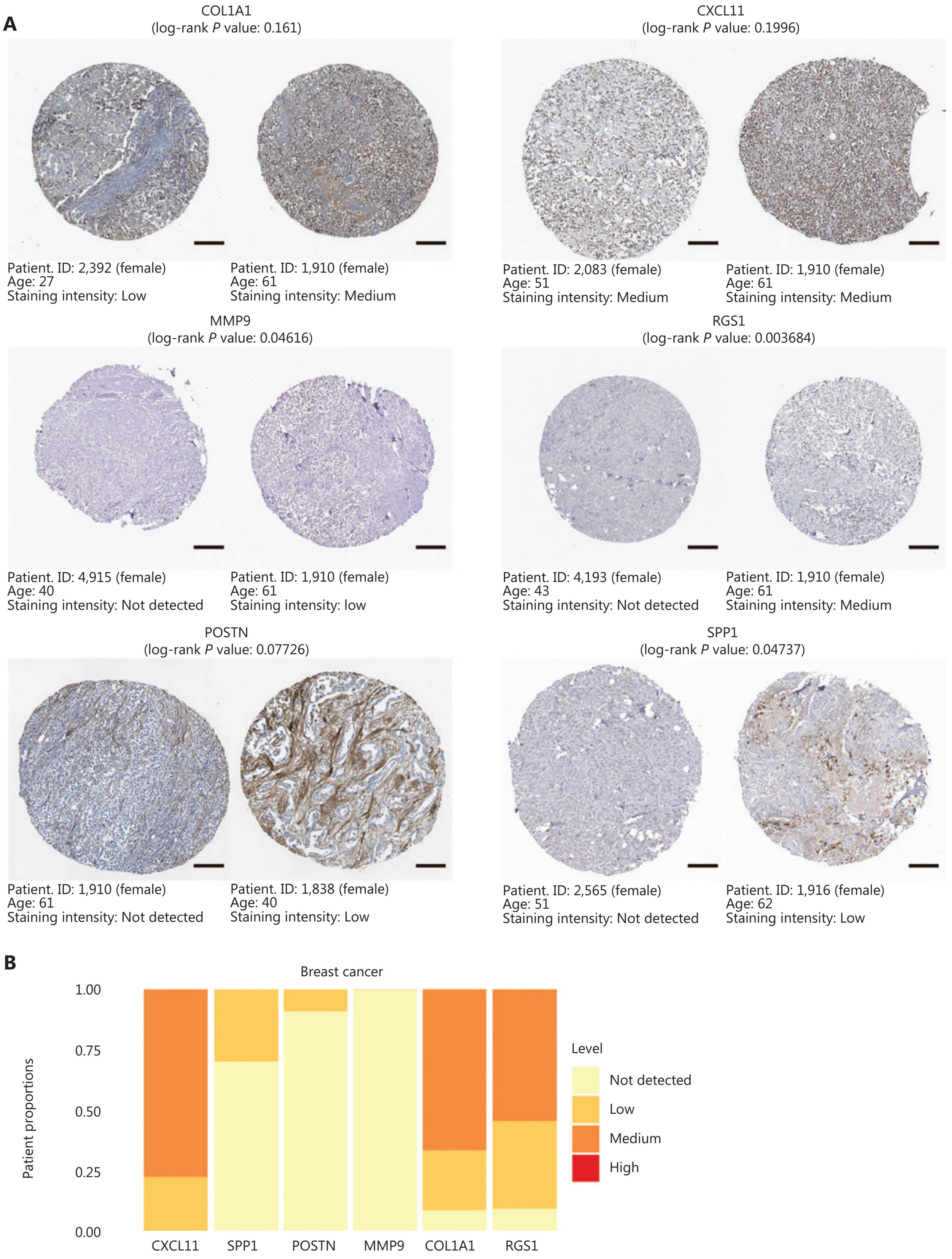

Figure S4 Protein expression analysis of hub genes in the HPA database. (A) COL1A1, CXCL11, MMP9, RGS1, POSTN, and SPP1 proteins were analyzed in breast cancer specimens (scale bar = 200 μm). (B) Statistical analysis for COL1A1, CXCL11, MMP9, RGS1, POSTN, and SPP1 proteins, on the basis of their expression intensity (not detected, low, moderate, and high).

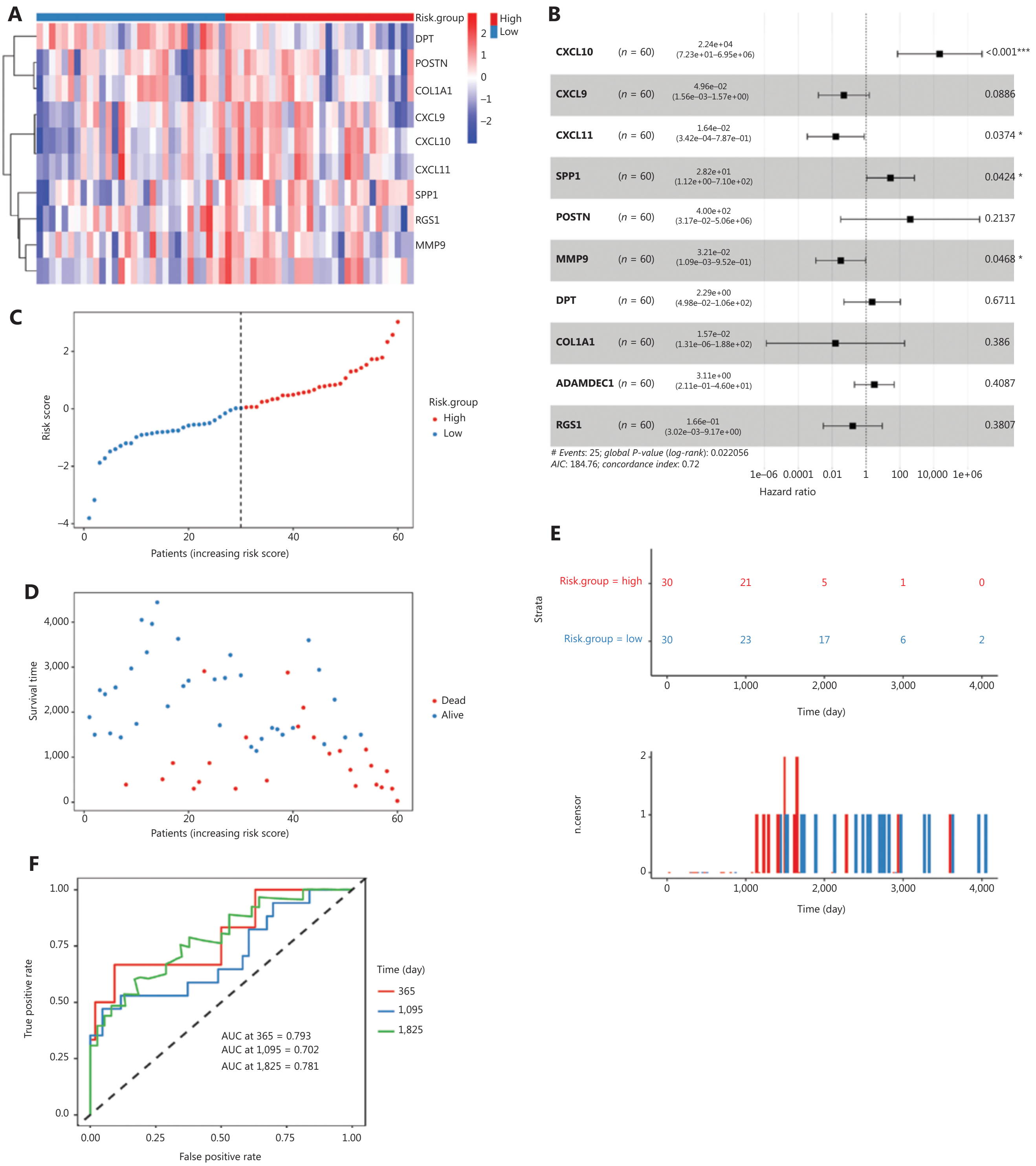

Figure S5 Construction and validation of the gene-based risk signature in dataset GSE37751. (A) Heatmap of the expression of the 10 hub genes in high- and low-risk groups. (B) Forest plot of the hazard ratios of the hub genes, by univariable Cox proportional hazards regression analysis. (C–D) Distribution of the risk score, survival time, and survival status. (E) Kaplan–Meier survival analysis. (F) ROC curve based on the hub-gene risk signature. Log-rank test: *P < 0.05, ***P < 0.001.
